# The effects of isotretinoin on serotonin: a prospective pilot study on acne patients^⋆^

**DOI:** 10.1016/j.abd.2021.02.011

**Published:** 2022-05-30

**Authors:** Adam P. Bray, Georgios Kravvas, Suzanne M. Skevington, Christopher R. Lovell

**Affiliations:** a Bristol Dermatology Centre, University Hospitals Bristol NHS Foundation Trust, Bristol, UK; b Manchester Centre for Health Psychology, School of Psychological Sciences, University of Manchester, Manchester, UK; c Kinghorn Dermatology Unit, Royal United Hospital, Bath, UK

Dear Editor,

Isotretinoin (13-cis retinoic acid) is a highly effective and commonly used treatment against acne vulgaris. Even though isotretinoin has been linked to low mood, depression, and suicidal ideation, a concrete link to adverse mood has not been proven yet, with some studies supporting this claim and others refuting it.^1^, ^2^, ^3^, ^4^, ^5^, ^6^

Since isotretinoin crosses the blood-brain barrier, a biological mechanism linking isotretinoin to depression is plausible.^3^, ^7^, ^8^, ^9^, ^10^ In the adult brain, receptors for retinoids are widely expressed, and isotretinoin could potentially regulate the expression of several neuronal genes.^8^, ^9^, ^10^

Serotonin (5-HT) is a widely recognized neurotransmitter and a key mediator of mood. Imbalances in serotonin levels have been linked to low mood and depression.^7^, ^8^ The present work’s objective was to pilot a study that tests the hypothesis that treatment with isotretinoin could lead to measurable changes in the concentration of key mood-related neurotransmitters. In order to do so, the authors performed a prospective cohort study assessing the effects of isotretinoin on the neurotransmitter’s’ serotonin (5-HT) and 5-HIAA (the main serotonin metabolite) in patients with acne vulgaris. Plasma levels of 5-HT and 5-HIAA were measured before, during, and after treatment with isotretinoin. Data were collected prior to treatment initiation, at two and four months of treatment, and one month after treatment cessation.

A total of 27 patients contributed blood prior to initiation of treatment. 24 and 22 patients had their serum measured at two and four months of treatment, respectively. Only four patients attended for blood testing one month after treatment cessation (Table 1).

**Table 1:** Total numbers of patients contributing data at each visit.

Time-points	5-HT & 5-HIAA
Baseline	27
2-months	24
4-months	22
1-month post-cessation	4

At the pre-treatment baseline, the authors found the following mean values: 5-HT 10.66, 5-HIAA 74.77.

Mean values at 2-months of treatment were found to be: 5-HT 9.64 (p = 0.633), 5-HIAA 44.31 (p = 0.082). At 4-months of isotretinoin therapy values had changed to: 5-HT 13.07 (p = 0.349), 5-HIAA 32.83 (p = 0.294). These findings did not represent statistically significant changes from the baseline.

The disappointingly low numbers of patients that attended the follow-up appointment at one month after cessation of treatment did not allow for a meaningful statistical analysis of post-isotretinoin effects.

A detailed documentation of the findings over time, as well as the changing ratios of HT to HA, are presented in Table 2.

**Table 2:** Nonparametric summary of the findings for 5-HT (HT), 5-HIAA (HA), and their ratio (HTHA) at baseline (0), 2-months (2), and 4-months (4) of treatment.

Test results over time	Number of patients	Min	Lower quartile	Median	Mean	Upper quartile	Max	Wilcoxon matched-pairs signed-ranks tests
5-HT_0	27	0.61	4.59	8.70	10.66	13.04	36.78	Baseline comparator
5-HT_2	24	1.26	5.89	7.60	9.64	11.54	28.32	p=0.633
5-HT_4	22	1.07	6.34	9.78	13.07	14.77	56.10	p=0.349
5-HIAA_0	26	7.05	14.77	26.23	74.77	74.86	502.30	Baseline comparator
5-HIAA_2	24	3.11	13.19	23.05	44.31	29.03	548.20	p=0.082
5-HIAA_4	22	2.26	8.50	25.67	32.83	41.12	143.70	p =0.294
HTHA_0	26	0.012	0.098	0.249	0.605	0.527	5.220	Baseline comparator
HTHA_2	21	0.015	0.203	0.312	0.475	0.496	1.803	p=0.523
HTHA_4	20	0.015	0.246	0.490	0.749	0.761	2.854	p=0.244

The relationship between isotretinoin and adverse mood changes is a highly debated aspect of isotretinoin therapy.

The authors did not find a significant link between treatment with isotretinoin and changes in the neurotransmitters 5-HT, 5-HIAA, or the ratio of the two. If a causal link is present between the two, it is likely mediated via a different neurochemical pathway.

Having said that, the pilot study has a small number of patients and significant attrition in follow-up. Therefore, even though it can act instructively, this work cannot fully support this claim. Larger, prospective, case-controlled studies will likely be required in order to address in detail the link between mood, isotretinoin, and neurotransmitters. The authors hope that this work will act as guidance and inspiration for such further undertakings.

## Financial support

None declared.

## Authors’ contributions

Adam P. Bray: Contributed to the design, recruitment, management, writing, and literature review associated with this study.

Georgios Kravvas: Contributed to the design, recruitment, management, writing, and literature review associated with this study.

Suzanne M. Skevington: Contributed to the design, recruitment, management, writing, and literature review associated with this study.

Christopher R. Lovell: Contributed to the design, recruitment, management, writing, and literature review associated with this study.

## Conflicts of interest

None declared.
